# Effect of Whole Blood Dietary Mineral Concentrations on Erythrocytes: Selenium, Manganese, and Chromium: NHANES Data

**DOI:** 10.3390/nu16213653

**Published:** 2024-10-27

**Authors:** Arthur M. Costa, Rebecca J. Sias, Sandra C. Fuchs

**Affiliations:** 1 Department of Chemistry, University of Chicago, Chicago, IL 60637, USA; arthurcosta@uchicago.edu; 2 Department of Biochemistry and Molecular Biology, University of Chicago, Chicago, IL 60637, USA; rjsias@uchicago.edu; 3 Postgraduate Program in Cardiology, School of Medicine, Universidade Federal do Rio Grande do Sul, Porto Alegre 90035-003, RS, Brazil

**Keywords:** chromium, selenium, manganese, erythrocytes, hematocrit, hemoglobin

## Abstract

Background: Selenium (Se), Manganese (Mn), and Chromium (Cr) are dietary minerals ingested from specific grains, vegetables, and animal meats. Prior research showed that these minerals affect animal erythrocyte health but have unknown effects on human red blood cells (RBCs) and hematology. This study evaluated the effects of these dietary minerals on RBC count, hematocrit, and hemoglobin. Methods: We conducted a cross-sectional analysis of 23,844 American participants from the 2015–2016 and 2017–2020 National Health and Nutrition Examination Survey. We evaluated sex, age, ethnicity, education, income, and smoking status as covariates. Linear regression analyses were conducted to evaluate the effect of Cr, Se, and Mn on RBC count, hematocrit, and hemoglobin levels. We employed subpopulation-exclusion regressions further to explore the distinct effects of mineral elevation and deficiency. Additional analyses were performed to examine the relationship between Mn and RBC hemoglobin, RBC distribution width, transferrin receptor concentrations, transferrin saturation, and serum iron levels to support the interpretation of our findings. Optimizable ensemble machine learning models were used to corroborate regression results. Results: Adjusting for covariates, Cr was inversely associated with RBC count (Exp(b) = 0.954), hemoglobin (Exp(b) = 0.868), and hematocrit (Exp(b) = 0.668). Conversely, Se was positively associated with RBC count (Exp(b) = 1.003), hemoglobin (Exp(b) = 1.012), and hematocrit (Exp(b) = 1.032). Mn was positively associated with RBC count (Exp(b) = 1.020) but inversely associated with hemoglobin (Exp(b) = 0.945) and hematocrit (Exp(b) = 0.891). Conclusions: Cr was harmful to RBC health in all subpopulations, whereas Se was protective. Mn appears to contribute to the development of microcytic anemia, but only in subjects with clinically elevated Mn levels. Thus, excessive consumption of foods and supplements rich in Cr and Mn may harm human erythrocyte health and hematology.

## 1. Introduction

Selenium (Se), manganese (Mn), and Chromium (Cr) are essential dietary minerals [1]. They act as important cofactors and bio-redox catalysts, but high concentrations of Cr, Se, and Mn are implicated in developing hemolytic anemia, selenosis, and manganism, respectively.

Chromium is a mineral in fruits, vegetables, and animal meats [2,3]. In erythrocytes, hexavalent chromium (Cr VI) is reduced and transported to the nuclear membrane, leading to genomic instability [4,5]. Selenium is an essential nutrient found in animal meats and is a residue in selenoproteins, which are responsible for reducing oxidized antioxidants such as glutathione peroxidase [6]. At low concentrations, Se seems to protect against stroke and cardiovascular disease. However, excessive Se can become incorporated into cysteine and methionine residues, leading to protein malformation [7,8,9]. Se daily consumption guidelines have recently been under scrutiny and are being lowered worldwide because of the risk of developing selenosis from excessive Se consumption [10]. Manganese is an essential nutrient found in vegetables and grains [11]. It serves as a vital cofactor for enzymes involved in carbohydrate and lipid metabolism [12], as well as for the enzyme manganese superoxide dismutase 2 (SOD2), which is a scavenger of reactive oxygen species (ROS) located in the mitochondria [13]. Exposure to excess Mn causes neurotoxicity and widespread cytotoxicity, primarily attributed to excessive Mn absorption by the mitochondria, leading to ROS accumulation and calcium uptake [14,15].

Animal studies provide evidence that Cr can hurt erythrocytes by forming ROS, but limited research has been conducted on humans [16]. Conversely, animal studies have shown that Se enhances and reinforces erythrocytes at low concentrations, decreasing osmotic fragility [17]. The effects of Mn on human and animal erythrocytes are largely unknown [18] but are worth investigating since Mn is known to modulate iron metabolism, which is highly relevant to erythrocytes [18,19]. The scarcity of human studies on the influence of these vital dietary minerals on red blood cells underscores a gap in our understanding of the relationship between nutrition and human biochemistry. This study, therefore, aims to investigate the effects of dietary exposure to Cr, Se, and Mn on erythrocytes in a representative population-based sample.

## 2. Methodology

We performed a comprehensive analysis using data obtained from the National Health and Nutrition Examination Survey (NHANES) conducted by the Center for Disease Control and Prevention (CDC) in 2015–2016 and 2017–2020 [20,21]. Our study focused on 23,844 American participants aged 18 years and older. All statistical tests, analyses, and descriptions (other than optimizable ensemble analyses) were performed using IBM’s Statistical Package for Social Sciences (SPSS, version 29.0.1.0, Chicago, IL, USA). Optimizable ensemble analyses were performed using MathWorks’ MATLAB (version 24.2.0, Natick, MA, USA).

Participants were interviewed at home to collect demographic, socioeconomic, and smoking data through surveys conducted from 2015 to 2020. Additionally, laboratory data collected from the CDC mobile examination center were analyzed, including hematological parameters and blood concentrations of Cr (in nmol/L), Se (in umol/L), and Mn (in nmol/L) levels. The data on Cr were only collected from participants aged 40 and above, a limitation of the NHANES sample that cannot be overcome. The information included sex, age, ethnicity (categorized as Mexican, Hispanic-non-Mexican, Non-Hispanic white, Non-Hispanic black, Non-Hispanic Asian, Multiracial or other), level of education (classified as less than 9th grade, 9–12th grade, high school graduate or GED, some college or AA degree, college graduate or above, and unknown), income (ratio of family income to poverty), and smoking (lifetime consumption of at least 100 cigarettes). Participants present in all analyses provided complete survey information and underwent blood tests to obtain Cr, Se, Mn, RBC, hematocrit, and hemoglobin levels. Participants with missing information for one or more variables were only excluded from analyses that required that parameter. Levels of normality for hematological parameters were obtained from the Mayo Clinic [22] and were used to describe the population. Weights were recalculated according to NHANES weighting procedures to ensure our analyses accurately represented the American population [23]. When describing the population, the sampling weights were normalized to have an average value of 1.00, as a standard practice previously employed in NHANES-based analyses [24].

Characteristics of the studied population are displayed as absolute numbers and percentages. The analysis tested for collinearity between the dietary minerals and hematocrit; hemoglobin, RBC, and residuals were analyzed for normality. We also performed collinearity tests between Cr, Mn, and Se themselves. All analyses were conducted using the entire study population.

Additional regression analyses were performed on sub-populations to test specific hypotheses. Regression analyses excluded participants with deficiencies in each trace element to isolate the effects of elevated levels from those of deficiency. Similarly, regressions were then performed excluding participants with elevated trace element levels to further distinguish between the effects of elevation and deficiency. The analyses used linear regressions with RBC count, hematocrit, and hemoglobin as dependent variables. Each regression analysis investigated the relationship between one trace element (Cr, Se, or Mn) and these hematological parameters.

Finally, to more precisely investigate how Mn impacted RBCs, we conducted further regression analyses between Mn levels and RBC hemoglobin, transferrin receptor levels (TfR), serum iron (Fe), and transferrin binding capacity. We also conducted a binary logistic regression between Mn levels and the incidence of recent anemia treatment to better understand the relationship between Mn levels and the incidence of anemia.

Additionally, simple linear regression models were used to explore the potential influence of sex, age, ethnicity, education, income, and smoking as confounding factors, setting the significance level at *p* < 0.2. Subsequently, we performed multiple regression analyses, accounting for confounding factors. Additionally, a term for the interaction between income and education was introduced because these confounders may be correlated with dietary mineral intake patterns. To overcome some degree of heteroskedasticity, we adopted heteroskedasticity-robust standard errors for calculating significance and effect sizes, enhancing the reliability and accuracy of our findings. To ensure the accuracy of our findings when considering non-linear relationships between mineral levels and hematological outcomes, we conducted optimizable ensemble (OE) machine learning analyses. In these analyses, we trained models to predict changes in hematological parameters using all metals and covariates simultaneously, which provided the highest predictive validity as measured by root mean squared error and R^2^. We did not consider sample weights due to limitations in the capability of even modern OE technology. However, the lack of weighting is not an issue because we used OE to evaluate the accuracy of our regression findings based on our sample data rather than using the OE to make broader claims about the effect of minerals on hematological parameters across the entire American population.

## 3. Results

### 3.1. Description of the Population

The NHANES sampling enrolled 23,844 individuals who provided information in the 2015–2016 or 2017–2020 surveys and a subset of 14,088 participants who also provided blood samples to assess whole blood hematological parameters.

Table 1 shows the characteristics of the weighted sample based on sex. There was an even distribution of sex, with participants averaging 48.27 ± 17.33 years old. Additionally, almost two-thirds of the participants were identified as non-Hispanic whites, which closely mirrors the demographic composition of the United States. Furthermore, most participants had completed at least a high school education and were non-smokers.

Table 2 shows that most participants had Cr deficiency, and none had clinically elevated levels. Most participants had normal Mn levels and elevated Se levels. Regardless of sex, most participants had average RBC counts, hemoglobin, and hematocrit levels. However, men experienced higher rates of elevation in each hematological parameter, while women showed higher rates of deficiency.

### 3.2. Regression Analysis Between Minerals and Blood Parameters

Table 3 presents the results of both unadjusted and adjusted linear regression models investigating the association between Cr, Mn, Se, and RBC count. Higher Cr levels were associated with a lower RBC count in the unadjusted model. After accounting for confounding variables and the interaction between income and education, the negative association between Cr and RBC count remained, although it was slightly attenuated. For each unit increase in Cr, the odds of having a higher RBC count decreased by approximately 4.6%, and after adjusting for covariates, this decrease was reduced to 3.7%.

Conversely, exposure to Mn was found to have a small positive association with RBC count. There was a 0.9% increase in the likelihood of a higher RBC count for each unit increase in Mn. This positive association became even stronger after accounting for confounding factors and the interaction between income and education, with the likelihood of having a higher RBC count increasing by 2.0% per unit increase in Mn. Se levels were positively associated with RBC count; for each unit increase in Se, the odds of the RBC count increased by 0.3%. The control for confounding factors did not significantly affect this association.

The analysis considered the potential interdependence between the dietary minerals Cr, Mn, and Se by using regression models to address multicollinearity. The collinearity diagnostics showed minimal multicollinearity between the independent variables (VIF < 1.400 and collinearity tolerance > 0.750). The highest collinearity was observed between income (VIF = 1.306) and education (VIF = 1.331). Still, the VIF values were low, indicating that multicollinearity was not significant enough to warrant excluding either variable as a covariate, especially considering the use of robust standard errors.

Table 4 presents the association between dietary minerals and hematocrit from unadjusted and adjusted linear regression models. The analyses for Cr showed a significant negative association with hematocrit, both in the unadjusted and adjusted analyses. In the former, it was found that for each unit increase in Cr, the odds of having a higher hematocrit decreased by approximately 33.2%. The negative association persisted after adjusting for confounders and the interaction term between income and education. However, the effect size was slightly attenuated, and it was observed that with each unit increase in Cr, the odds of having a higher hematocrit decreased by approximately 32.4%.

In the unadjusted analysis, exposure to Mn exhibited a strong negative association with increased hematocrit, where higher Mn levels were linked to a 10.9% decrease in the odds of having a higher hematocrit. Adjusting for confounding factors substantially decreased the magnitude of this association, resulting in only a 2.5% decrease in hematocrit odds with increased Mn levels. This adjustment suggests that socioeconomic factors, along with other confounders, played an important role in modifying the relationship between Mn and hematocrit.

For each unit increase in Se, the odds of having a higher hematocrit increased by approximately 3.2%. After adjusting for confounding factors and the interaction term between income and education, the positive association remained statistically significant, although the effect size was slightly reduced. Specifically, for each unit increase in Se, the odds of having a higher hematocrit increased by about 2.4%.

Table 5 presents the outcomes from both unadjusted and adjusted linear regression models investigating the association between Cr, Mn, Se, and hemoglobin. It shows a robust inverse association between Cr and hemoglobin. Levels. It showed that for every unit increase in Cr, hemoglobin decreased by around 13.2%. After accounting for confounding factors and the interaction between income and education, the decrease in hemoglobin remained nearly the same at 13.3%. This suggests that socioeconomic factors did not significantly impact the relationship between Cr and hemoglobin. The association between Mn and hemoglobin level was found to be inverse. In the unadjusted analysis, it was observed that for each unit increase in Mn, hemoglobin decreased by about 5.5%. After adjusting for confounding factors and the interaction term between income and education, the association attenuated, and a unit increase in Mn was now associated with a 2.4% decrease in hemoglobin.

Table 5 also shows a positive association between Se and hemoglobin levels. Higher Se levels were linked to slightly higher hemoglobin levels. After adjusting for confounding factors and including the interaction term between income and education, the Exp(Beta) decreased from 1.012 to 1.009. This indicates that for each unit increase in Se, hemoglobin, which initially increased by 1.2%, now increased by 0.9%. This slight decrease in the positive association suggests that other factors explained some of the variation, although Se still had a small positive effect on hemoglobin levels.

### 3.3. Basic Interpretation of Regression Results

Excluding participants with Cr deficiencies reduced the effect sizes (|1 − Exp(b)|) on RBC count, hematocrit, and hemoglobin outcomes. Still, the inverse relationship between Cr levels and each blood parameter remained consistent and significant (Supplementary Tables S1–S3). Considering that we observed no subjects with elevated Cr levels (Table 2), the reduced effect after excluding Cr-deficient subjects suggests that the negative association is most vital in subjects with normal Cr levels. There was, however, one unusual finding when deficient subjects were excluded: Cr showed a slight positive association with hematocrit when not accounting for covariates (Exp(b) = 1.007; *p* < 0.001). When covariates were considered, this association changed and more closely resembled the results in Table 4 (Exp(b) = 0.970; *p* < 0.001).

Both the models excluding Mn-deficient and those excluding elevated-Mn subjects had similar effects on RBC count, as reported in Table 3 (Tables S1 and S4). The fact that the association persisted despite removing deficient subjects suggests that increasing Mn levels, even within the normal range, continue to elevate RBC count. Similarly, models excluding Mn-deficient subjects did not show a significantly different effect on hematocrit or hemoglobin compared to the results in Table 4 and Table 5 (Tables S2 and S3). However, the models that excluded subjects with elevated Mn levels showed significant positive associations between Mn and both hemoglobin and hematocrit (adjusted models: Exp(b) = 1.154, *p* < 0.001; Exp(b) = 1.044, *p* < 0.001), while Table 4 and Table 5 reported negative associations between Mn and both hemoglobin and hematocrit (Tables S5 and S6). This finding suggests that Mn elevation may decrease hemoglobin and hematocrit levels. We considered that the simultaneous increase in RBC count alongside decreases in hemoglobin and hematocrit (in Mn-elevated subjects) suggests a reduction in RBC volume and conducted a further regression analysis between Mn and RBC volume, revealing a marked negative association (Exp(b) = 0.645, *p* < 0.001). Additionally, we performed another regression to analyze the effect of Mn on RBC distribution width, uncovering a significant positive association (Exp(b) = 1.125, *p* < 0.001).

Exploring the effects of Mn on TfR to explain how Mn could affect downstream hemoglobin and hematocrit levels, we conducted a further regression analysis between Mn and TfR levels, which showed a positive association (Exp(b) = 1.252, *p* < 0.001). Then, exploring the effect of Mn on iron metabolism as a mechanistic alternative, we conducted regression analyses between Mn and serum iron (Exp(b) = 0.175, *p* < 0.001), transferrin saturation, accounting for serum Fe as a covariate (Exp(b) = 0.452, *p* < 0.001), and RBC hemoglobin (Exp(b) = 0.834, *p* < 0.001), all of which showed significant negative associations. Finally, exploring the effect of Mn on the incidence of anemia, we found a significant positive association between Mn levels and being recently treated for anemia (Exp(b) = 1.095, *p* < 0.001).

Both models excluding Se deficient subjects (Tables S1–S3) and those excluding subjects with elevated Se (Tables S4–S6) showed similar effects on RBC count, hemoglobin, and hematocrit as those reported in Table 3, Table 4 and Table 5, but all models excluding subjects with elevated Se did have slightly higher effect sizes (Exp(b) range = 1.006–1.071) than those reported in Table 3, Table 4 and Table 5 (Exp(b) range = 1.003–1.032). Regardless, the positive associations remained significant even after excluding subjects with Se deficiency or elevation, indicating that the effects of Se on these hematological parameters are not due to clinical Se deficiency or elevation.

### 3.4. Optimizable Ensemble Machine Learning Analyses

The OE modeling analyzed the relationship between levels of each mineral and hematological outcomes and allowed us to plot partial dependence. The models effectively captured the nonlinear effects of minerals on hematological outcomes and showed moderate predictive significance (RBC counts R^2^ = 0.25; hemoglobin R^2^ = 0.45; hematocrit R^2^ = 0.44). All R^2^ values and partial dependence plots are from the testing (rather than training) data. Supplementary Figures S1–S9 show the partial dependence of RBC count, hemoglobin, and hematocrit on Cr, Mn, and Se. All partial dependence plots confirmed the general trends identified through our linear regression analyses. Chromium consistently showed negative associations with RBC count, hemoglobin, and hematocrit, which agrees with the regression results above (Figures S1, S4 and S7). Interestingly, beyond approximately 4 μg/L, further increases in Cr did not lead to additional decreases in RBC count, hemoglobin, or hematocrit in the sampled population. Manganese exhibited consistently positive associations with RBC count (Figure S2) and parabolic associations with hemoglobin and hematocrit, centered at approximately 12 μg/L, which is at the upper end of the normal range (4–15 μg/L) (Figures S5 and S8). These results agree with our regression results, which indicated a positive association between Mn and RBC count, a positive association with hemoglobin and hematocrit when subjects >15 μg/L Mn were excluded, and a negative association with hemoglobin and hematocrit when subjects >15 μg/L were included. Selenium consistently showed positive associations with RBC count, hemoglobin, and hematocrit, aligning with the regression results above (Figures S3, S6 and S9).

## 4. Discussion

The findings of negative associations between Cr and RBC, hematocrit, and hemoglobin are aligned with previous literature, which has demonstrated causative inverse relationships between blood Cr levels and hematocrit, hemoglobin, and RBC counts in animal studies [25,26]. In vitro studies show that Cr III does not permeate significantly into human erythrocytes, but Cr VI does [27]. After Cr VI enters erythrocytes, it binds to hemoglobin and peptides such as glutathione, forming stable complexes that sequester the Cr in the erythrocyte for an extended period [27]. Cr forming stable complexes with glutathione decreases RBC’s antioxidant load. Furthermore, Cr VI is readily reduced to Cr V and then Cr III in erythrocytes [5]. This reduction pathway creates ROS byproducts. Cr being sequestered in erythrocytes for long periods, which decreases antioxidant capacity and directly generates ROS (leading to extensive damage), helps explain the negative associations between Cr and RBC count, hemoglobin, and hematocrit [5,28].

The Agency for Toxic Substances and Disease Registry has highlighted the potential adverse impact of Cr exposure on erythrocyte health, identifying it as a contributor to microcytic hypochromic anemia [29]. In accordance with this, our findings suggest that Cr may significantly negatively affect RBC health. However, the generalizability of these findings is limited because the analyzed population lacked blood Cr data for participants younger than 40 years. Furthermore, our population lacked any participants with a clinical excess in Cr, further limiting the generalizability of our findings. Thus, significant further investigation is needed into the mechanisms and effects of Cr on human erythrocytes and hematology.

The most plausible explanation for the positive associations we found between Se and RBC, hemoglobin, and hematocrit lies in the role of selenoproteins, which reduce hydroperoxides and ROS that can damage RBC cell membranes, hemoglobin, and membrane proteins [17,30,31]. Given that selenoproteins are needed to reduce hydroperoxide levels, lower Se (and thus selenoprotein) levels lead to hydroperoxide accumulation, leading to widespread damage (including RBCs and RBC progenitors). As the central role of Se is as a cofactor in selenoproteins, their antioxidizing properties are likely the most critical factors contributing to the positive association seen between Se and hemoglobin, hematocrit, and RBC counts. This finding aligns with existing animal studies, underscoring the importance of adequate Se concentrations for erythrocyte membrane health, hemoglobin levels, and the differentiation of hematopoietic stem cells into erythrocytes [17,31].

The positive relationship between Mn levels and RBC count we observed is likely due to the role of SOD2. SOD2 levels are inversely associated with ROS concentrations in RBC precursors; therefore, higher blood Mn levels may increase SOD2 levels, reducing oxidative stress on RBC progenitors and resulting in a more significant proportion of progenitors differentiating into mature erythrocytes [12,32,33]. However, our finding of negative associations between Mn and hemoglobin and hematocrit when subjects with clinical Mn elevation were included (despite the positive association between Mn and RBC) merited further mechanistic exploration. We first considered Mn’s disruptive effect on Iron Regulatory Protein (IRP) activity [34]. IRPs bind to TfR mRNA sequences and other iron-responsive elements (IREs). Greater TfR mRNA stability afforded by IRP binding leads to more extensive TfR production and greater Fe uptake downstream because of TfR’s central role in erythrocytic Fe uptake [5]. This idea is further supported by previous findings of a negative association between Mn and TfR levels [35]. Thus, we hypothesized that Mn interference with IRP activity could decrease TfR levels and, therefore, decrease Fe uptake, helping to explain the decreases in hemoglobin and hematocrit levels we associated with increased Mn levels. To test this, we conducted a regression analysis between Mn and blood TfR levels and found a positive relationship, rejecting our hypothesis.

We then considered the alternative hypothesis that Mn’s effects on erythrocytes were primarily mediated by competitive inhibition of the divalent metal transporter (DMT1) [33,36]. DMT1 has a higher affinity for Mn than Fe, and in vitro models have shown that cellular incubation with Mn leads to decreased Fe uptake via DMT1 [33]. Furthermore, erythrocytes with mutated DMT1s have been shown to exhibit signs of microcytic anemia despite having adequate Fe levels in surrounding media, showing the importance of DMT1 to erythrocytic Fe uptake [33]. Mn-mediated competitive inhibition of DMT1 thus presents a plausible mechanism through which Mn would lead to decreased intracellular Fe levels and a subsequent decrease in hemoglobin and hematocrit.

Exploring these effects, we found a negative association between Mn and serum Fe consistent with the literature [33]. Investigating whether Mn was altering Fe uptake (as suggested by the DMT1 inhibition mechanism), we found negative associations between Mn and RBC hemoglobin and RBC volume, and a positive association between Mn and RBC distribution width. These findings (i.e., decreased RBC hemoglobin and volume with concurrently increased distribution width) are characteristic of microcytic anemia and are seen in patients with dysfunctional DMT1 [37,38,39]. To investigate the association between Mn and anemia, we conducted a regression between Mn and the incidence of recent anemia treatment (in the three weeks preceding data collection). We found a significant positive association, suggesting once more that Mn may contribute to the incidence of anemia. However, the generalizability of this finding is limited because it does not account for differential disposition to seeking treatment or socioeconomic conditions affecting treatment availability. Regardless, although Mn increases RBC count, it decreases hemoglobin and hematocrit levels and appears to be a potentially significant contributor to the onset of microcytic anemia because it inhibits DMT1-mediated Fe transport. Still, more research is needed to describe this association and its mechanism further.

These findings indicate that socioeconomic factors—income and education—may modify the impact of trace minerals, particularly Cr and Mn, on RBC count. While higher chromium levels were consistently associated with lower RBC count, Mn had a stronger positive association with RBC count. The positive association of Se with RBC count remained stable, with minimal impact from confounders.

The results also indicate that levels of trace minerals are associated with variations in hematocrit, and socioeconomic factors influence these relationships. Chromium and Mn showed a consistent negative association with hematocrit (when elevated subjects were included in Mn analyses), though the effect sizes were slightly reduced after adjusting for confounding factors. In contrast, Se maintained a positive association with hematocrit, and although the effect size decreased somewhat after adjustment, the relationship remained significant. These findings underscore the need to consider socioeconomic factors when evaluating the impact of dietary minerals on hematocrit levels.

After adjusting for confounding factors, the inverse and nearly unchanged association between Cr and hemoglobin suggests that socioeconomic factors had minimal influence. In contrast, the inverse association of Mn with hemoglobin (when elevated subjects were included) was halved after adjustment, indicating that socioeconomic factors may partly explain this relationship. The positive association of Se with hemoglobin decreased slightly, retaining a small positive effect on hemoglobin levels.

Our study has some limitations, which should be taken into account in the interpretation of the results. The absence of participants with clinically elevated Cr levels precluded analyzing the effects of overexposure. However, the NHANES provides a representative sample of the US population, and the lack of individuals with elevated Cr levels in a sample of over 23,000 reflects the real-world distribution of Cr exposure within the US. Although this limitation prevented us from assessing the effects of elevated Cr levels, the analysis remained robust for understanding its impact within low and normal ranges. Additionally, the sample included individuals with elevated levels of selenium and manganese, other essential minerals.

The data on Cr were only collected from participants aged 40 and above due to constraints in the NHANES lab sample. This limitation may restrict the generalizability of our findings to younger populations. While chromium may be involved in metabolic processes that could indirectly impact blood parameters, there is minimal direct evidence of its effect on RBC count, hematocrit, and hemoglobin in individuals under 40 with diabetes mellitus, cardiovascular diseases, and depression. Future research should include younger populations to understand age-related differences in dietary behaviors and exposure to chromium better.

The measurement of Cr had limited sensitivity, with some participants having levels below the detection limit. This has been addressed in the analysis. These values were set to the minimum detectable level and multiplied by √2, which required us to apply heteroskedasticity-robust standard errors in the regression models.

Similarly, only a few participants were clinically deficient in Se and Mn, limiting the extent to which our analyses could account for addressing the effect of deficiencies. Despite these limitations, we conducted analyses that provided insight into the impact of comparatively lower or higher dietary mineral levels on RBC count, hematocrit, and hemoglobin. We also performed subgroup analyses, allowing us to examine the effects of mineral deficiency and elevation. A final limitation is that our OE models are unweighted, preventing them from being provably reliable predictors for these effects in the general American population. This is not a problem, however, as they were used strictly to assess the reliability of our regression models by evaluating the isolated nonlinear effects of mineral levels on hematological outcomes in the sample population.

## 5. Conclusions

Our present findings suggest that excess Cr may adversely affect erythrocyte health, but further research with younger subjects and subjects with clinical Cr elevations is needed. The outcomes of our study also suggest that adequate Se levels may be an essential factor in maintaining erythrocyte health. Furthermore, our findings indicate that Mn deficiency may impede proper Fe metabolism. Moreover, we showed that Mn decreased RBC volume, RBC hemoglobin, and whole blood hemoglobin while increasing RBC distribution width, suggesting that it may be a significant contributing factor toward the development of microcytic anemia. Further in vitro and in vivo research is needed to understand the complete proteomic changes incurred to erythrocytes by exposure to these dietary minerals and the subsequent downstream effects of those changes.

## Figures and Tables

**Table 1:** Characteristics of the studied population according to sex [*n* (%)].

		Total	Men	Women	*p* Value *
Age (years)	20–29	2701 (18.4)	1352 (19.1)	1349 (17.7)	<0.001
	30–39	2603 (17.7)	1283 (18.1)	1320 (17.3)	
	40–49	2458 (16.7)	1212 (17.1)	1246 (16.3)	
	50–59	2641 (18)	1273 (18.0)	1368 (17.9)	
	60–69	2263 (15.4)	1072 (15.2)	1191 (15.6)	
	70–79	1350 (9.2)	607 (8.6)	743 (9.7)	
	≥80	684 (4.7)	273 (3.9)	411 (5.4)	
Ethnicity	Mexican	1244 (8.5)	631 (8.9)	613 (8.0)	<0.001
	Hispanic-non-Mexican	1072 (7.3)	515 (7.3)	557 (7.3)	
	Non-Hispanic white	9256 (63)	4466 (63.2)	4790 (62.8)	
	Non-Hispanic black	1681 (11.4)	751 (10.6)	930 (12.2)	
	Non-Hispanic Asian	870 (5.9)	401 (5.7)	469 (6.2)	
	Multiracial or other	577 (3.9)	308 (4.4)	269 (3.5)	
Education	<9th grade	657 (4.5)	323 (4.6)	334 (4.4)	<0.001
	9–12th grade	1123 (7.6)	597 (8.5)	526 (6.9)	
	High school graduate or GED	3678 (25)	1861 (26.3)	1817 (23.8)	
	Some college or AA degree	4566 (31.1)	2088 (29.5)	2478 (32.5)	
	College graduate or above	4666 (31.7)	2199 (31.1)	2467 (32.4)	
Smoked ≥100 cigarettes in lifetime	Yes	6297 (42.8)	3571 (50.5)	2726 (35.8)	<0.001
	No	8395 (57.1)	3496 (49.5)	4899 (64.2)	

* Chi-square test.

**Table 2:** Whole blood characteristics of the sample population [*n* (%)].

	Normal Range	Total *n*	Deficient *n*	Elevated *n*
Chromium	0.7–28 μg/L	9040	8377 (92.7)	0 (0.0)
Manganese	4–15 μg/L	11,915	68 (0.6)	792 (6.6)
Selenium	120–160 μg/L	11,915	17 (0.1)	10,688 (89.7)
RBC count (male)	4.35–5.65 million cells/mcl	6728	483 (7.2)	367 (5.5)
RBC count (female)	3.92–5.13 million cells/mcl	7320	389 (5.3)	388 (5.3)
Hemoglobin (male)	13.2–16.6 g/dL	6728	408 (6.1)	440 (6.5)
Hemoglobin (female)	11.6–15 g/dL	7320	476 (6.5)	461 (6.3)
Hematocrit (male)	38.3–48.6%	6768	294 (4.3)	590 (8.7)
Hematocrit (female)	35.5–44.9%	7320	538 (7.3)	389 (5.3)

**Table 3:** Effect of dietary minerals: Chromium, Manganese, and Selenium on RBC count.

	*n*	Unstandardized Beta	Error	Exp(B) (95%CI)	*p* Value
Chromium level (μg/L)	9066	−0.047	8.19 × 10^−5^	0.954 (0.954–0.954)	<0.001
Adjusted chromium (μg/L) *	7918	−0.038	7.80 × 10^−5^	0.963 (0.962–0.963)	<0.001
Manganese level (μg/L)	10,686	0.009	1.14 × 10^−5^	1.009 (1.009–1.009)	<0.001
Adjusted manganese (μg/L) *	9315	0.02	1.20 × 10^−5^	1.020 (1.020–1.020)	<0.001
Selenium level (μg/L)	10,686	0.003	1.58 × 10^−6^	1.003 (1.003–1.003)	<0.001
Adjusted selenium (μg/L) *	9315	0.003	1.47 × 10^−6^	1.003 (1.003–1.003)	<0.001

Linear regression model with robust standard error. * Exp(B) 95%CI adjusted for age, sex, ethnicity, income, education, smoking, and income × education.

**Table 4:** Effect of dietary minerals: Chromium, Manganese, and Selenium on hematocrit.

	*n*	Unstandardized Beta	Error	Exp(B) (95%CI)	*p* Value
Chromium level (μg/L)	9066	−0.403	7.00 × 10^−4^	0.668 (0.667–0.669)	<0.001
Adjusted chromium (μg/L) *	7918	−0.392	8.00 × 10^−4^	0.676 (0.675–0.677)	<0.001
Manganese level (μg/L)	10,686	−0.116	1.00 × 10^−4^	0.891 (0.891–0.891)	<0.001
Adjusted manganese (μg/L) *	9315	−0.026	1.00 × 10^−4^	0.975 (0.975–0.975)	<0.001
Selenium level (μg/L)	10,686	0.032	1.29 × 10^−5^	1.032 (1.032–1.032)	<0.001
Adjusted selenium (μg/L) *	9315	0.024	1.15 × 10^−5^	1.024 (1.024–1.024)	<0.001

Linear regression model with robust standard error. * Exp(B) 95%CI adjusted for age, sex, ethnicity, income, education, smoking, and income × education.

**Table 5:** Effect of dietary minerals: Chromium, Manganese, and Selenium on hemoglobin.

	*n*	Unstandardized Beta	Error	Exp(B) (95%CI)	*p* Value
Chromium level (μg/L)	9066	−0.141	3.00 × 10^−4^	0.868 (0.868–0.869)	<0.001
Adjusted chromium (μg/L) *	7918	−0.142	3.00 × 10^−4^	0.867 (0.867–0.868)	<0.001
Manganese level (μg/L)	10,686	−0.056	4.18 × 10^−5^	0.945 (0.945–0.945)	<0.001
Adjusted manganese (μg/L) *	9315	−0.024	4.20 × 10^−5^	0.976 (0.976–0.976)	<0.001
Selenium level (μg/L)	10,686	0.012	4.70 × 10^−6^	1.012 (1.012–1.012)	<0.001
Adjusted selenium (μg/L) *	9315	0.009	4.10 × 10^−6^	1.009 (1.009–1.009)	<0.001

Linear regression model with robust standard error. * Exp(B) 95%CI: adjusted for age, sex, ethnicity, income, education, smoking, and income × education.

## Data Availability

The datasets analyzed in this study are available in the 2015–2016 and 2017–2020 NHANES repositories, https://wwwn.cdc.gov/nchs/nhanes/continuousnhanes/default.aspx?BeginYear=2015, accessed on 25 September 2024 and https://wwwn.cdc.gov/nchs/nhanes/continuousnhanes/default.aspx?Cycle=2017-2020, accessed on 25 September 2024.

## Supplementary Materials

The following supporting information can be downloaded at: https://www.mdpi.com/article/10.3390/nu16213653/s1, Figure S1: Partial Dependence of RBC count on chromium; Figure S2. Partial Dependence of RBC count on manganese; Figure S3. Partial Dependence of RBC count on selenium; Figure S4. Partial Dependence of hemoglobin on chromium; Figure S5. Partial Dependence of hemoglobin on manganese; Figure S6. Partial Dependence of hemoglobin on selenium; Figure S7. Partial Dependence of hematocrit on chromium; Figure S8. Partial Dependence of hematocrit on manganese; Figure S9. Partial Dependence of hematocrit on selenium; Table S1: Effect of trace elements on elevated and normal subjects: Chromium, Manganese, and Selenium on RBC Count; Table S2: Effect of trace elements on elevated and normal subjects: Chromium, Manganese, and Selenium on Hematocrit; Table S3. Effect of trace elements on elevated and normal subjects: Chromium, Manganese, and Selenium on Hemoglobin; Table S4. Effect of trace elements on deficient and normal subjects: Chromium, Manganese, and Selenium on RBC Count; Table S5. Effect of trace elements on deficient and normal subjects: Chromium, Manganese, and Selenium on Hematocrit; Table S6. Effect of trace elements on deficient and normal subjects: Chromium, Manganese, and Selenium on Hemoglobin.
