# Supplementary material for: Effect of Whole Blood Dietary Mineral Concentrations on Erythrocytes: Selenium, Manganese, and Chromium: NHANES Data
Source: Nutrients. 2024 Oct 27;16(21):3653. doi: 10.3390/nu16213653 (PMC11547540; doi:10.3390/nu16213653)
Supplement: Supplementary file 1 [file nutrients-16-03653-s001.zip › nutrients-3274668-supplementary.pdf]

**Table S1**. Effect of trace elements on elevated and normal subjects: Chromium, Manganese, and Selenium on RBC Count

| | n | Unstandardized beta | Error | Exp(B) 95%CI | P value |
|---|---|---|---|---|---|
| Chromium level (ug/L) | 655 | -0.015 | 1.00E-04 | 0.986 (0.985-0.986) | < 0.001 |
| Adjusted chromium (ug/L)* | 573 | -0.02 | 8.63E-05 | 0.981 (0.980-0.981) | < 0.001 |
| Manganese level (ug/L) | 10591 | 0.008 | 1.13E-05 | 1.008 (1.008-1.008) | <0.001 |
| Adjusted manganese (ug/L)* | 9238 | 0.019 | 1.19E-05 | 1.019 (1.019-1.019) | <0.001 |
| Selenium level (ug/L) | 10662 | 0.003 | 1.58E-06 | 1.003 (1.003-1.003) | <0.001 |
| Adjusted selenium (ug/L)* | 9295 | 0.002 | 1.47E-06 | 1.003 (1.002-1.003) | <0.001 |

Linear regression model with robust standard error
* Linear regression model adjusted for age, sex, ethnicity, income, education, smoking, and income*education

**Table S2**. Effect of trace elements on elevated and normal subjects: Chromium, Manganese, and Selenium on Hematocrit

| | n | Unstandardized beta | Error | Exp(B) 95%CI | P value |
|---|---|---|---|---|---|
| Chromium level (ug/L) | 655 | -0.052 | 0.0008 | 0.949 (0.948-0.951) | < 0.001 |
| Adjusted chromium (ug/L)* | 573 | -0.138 | 0.0005 | 0.871 (0.870-0.872) | < 0.001 |
| Manganese level (ug/L) | 10591 | -0.126 | 0.0001 | 0.881 (0.881-0.882) | <0.001 |
| Adjusted manganese (ug/L)* | 9238 | -0.035 | 0.0001 | 0.966 (0.966-0.966) | <0.001 |
| Selenium level (ug/L) | 10662 | 0.032 | 1.29E-05 | 1.032 (1.032-1.032) | <0.001 |
| Adjusted selenium (ug/L)* | 9295 | 0.023 | 1.16E-05 | 1.024 (1.024-1.024) | <0.001 |

Linear regression model with robust standard error
* Linear regression model adjusted for age, sex, ethnicity, income, education, smoking, and income*education

**Table S3**. Effect of trace elements on elevated and normal subjects: Chromium, Manganese, and Selenium on Hemoglobin

| | n | Unstandardized beta | Error | Exp(B) 95%CI | P value |
|---|---|---|---|---|---|
| Chromium level (ug/L) | 655 | 0.007 | 0.0003 | 1.007 (1.006-1.007) | < 0.001 |
| Adjusted chromium (ug/L)* | 573 | -0.03 | 0.0002 | 0.970 (0.970-0.971) | < 0.001 |
| Manganese level (ug/L) | 10591 | -0.061 | 4.17E-05 | 0.941 (0.941-0.941) | <0.001 |
| Adjusted manganese (ug/L)* | 9238 | -0.028 | 4.17E-05 | 0.973 (0.972-0.973) | <0.001 |
| Selenium level (ug/L) | 10662 | 0.012 | 4.72E-06 | 1.012 (1.012-1.012) | <0.001 |
| Adjusted selenium (ug/L)* | 9295 | 0.009 | 4.12E-06 | 1.009 (1.009-1.009) | <0.001 |

Linear regression model with robust standard error
* Linear regression model adjusted for age, sex, ethnicity, income, education, smoking, and income*education

**Table S4**. Effect of trace elements on deficient and normal subjects: Chromium, Manganese, and Selenium on RBC Count

| | n | Unstandardized beta | Error | Exp(B) 95%CI | P value |
|---|---|---|---|---|---|
| Chromium level (ug/L) | 9066 | -0.047 | 8.19E-05 | 0.954 (0.954-0.954) | < 0.001 |
| Adjusted chromium (ug/L)* | 7918 | -0.038 | 7.80E-05 | 0.963 (0.962-0.963) | < 0.001 |
| Manganese level (ug/L) | 9833 | 0.017 | 1.59E-05 | 1.017 (1.017-1.017) | <0.001 |
| Adjusted manganese (ug/L)* | 8574 | 0.03 | 1.48E-05 | 1.030 (1.030-1.030) | <0.001 |
| Selenium level (ug/L) | 1349 | 0.008 | 1.19E-05 | 1.008 (1.008-1.008) | <0.001 |
| Adjusted selenium (ug/L)* | 1149 | 0.006 | 1.13E-05 | 1.006 (1.006-1.006) | <0.001 |

Linear regression model with robust standard error
* Linear regression model adjusted for age, sex, ethnicity, income, education, smoking, and income*education

**Table S5**. Effect of trace elements on deficient and normal subjects: Chromium, Manganese, and Selenium on Hematocrit

| | n | Unstandardized beta | Error | Exp(B) 95%CI | P value |
|---|---|---|---|---|---|
| Chromium level (ug/L) | 9066 | -0.403 | 7.00E-04 | 0.668 (0.667-0.669) | < 0.001 |
| Adjusted chromium (ug/L)* | 7918 | -0.392 | 8.00E-04 | 0.676 (0.675-0.677) | < 0.001 |
| Manganese level (ug/L) | 9833 | 0.023 | 0.0001 | 1.023 (1.023-1.024) | < 0.001 |
| Adjusted manganese (ug/L)* | 8574 | 0.143 | 0.0001 | 1.154 (1.154-1.154) | < 0.001 |
| Selenium level (ug/L) | 1349 | 0.068 | 0.0001 | 1.071 (1.071-1.071) | < 0.001 |
| Adjusted selenium (ug/L)* | 1149 | 0.043 | 9.63E-05 | 1.044 (1.043-1.044) | < 0.001 |

Linear regression model with robust standard error
* Linear regression model adjusted for age, sex, ethnicity, income, education, smoking, and income*education

**Table S6**. Effect of trace elements on deficient and normal subjects: Chromium, Manganese, and Selenium on Hemoglobin

| | n | Unstandardized beta | Error | Exp(B) 95%CI | P value |
|---|---|---|---|---|---|
| Chromium level (ug/L) | 9066 | -0.141 | 3.00E-04 | 0.868 (0.868-0.869) | < 0.001 |
| Adjusted chromium (ug/L)* | 7918 | -0.142 | 3.00E-04 | 0.868 (0.867-0.868) | < 0.001 |
| Manganese level (ug/L) | 9833 | 0.001 | 4.84E-05 | 1.001 (1.001-1.001) | <0.001 |
| Adjusted manganese (ug/L)* | 8574 | 0.043 | 4.27E-05 | 1.044 (1.044-1.044) | <0.001 |
| Selenium level (ug/L) | 1349 | 0.028 | 3.86E-05 | 1.029 (1.029-1.029) | <0.001 |
| Adjusted selenium (ug/L)* | 1149 | 0.017 | 3.46E-05 | 1.018 (1.018-1.018) | <0.001 |

Linear regression model with robust standard error
* Linear regression model adjusted for age, sex, ethnicity, income, education, smoking, and income*education

**Figure S1.** Partial Dependence of RBC count on chromium

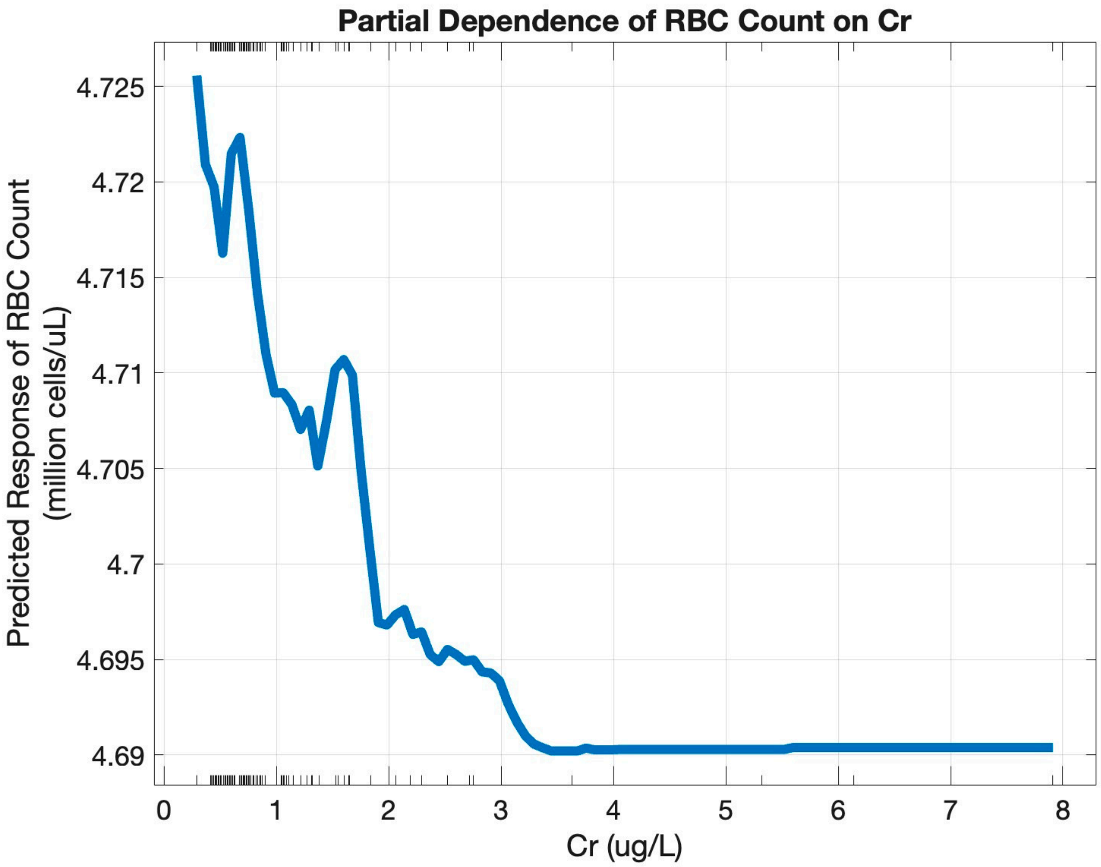


Optimizable ensemble model

Trained with Cr, Mn, Se, age, sex, ethnicity, income, education, and smoking data

**Figure S2.** Partial Dependence of RBC count on manganese

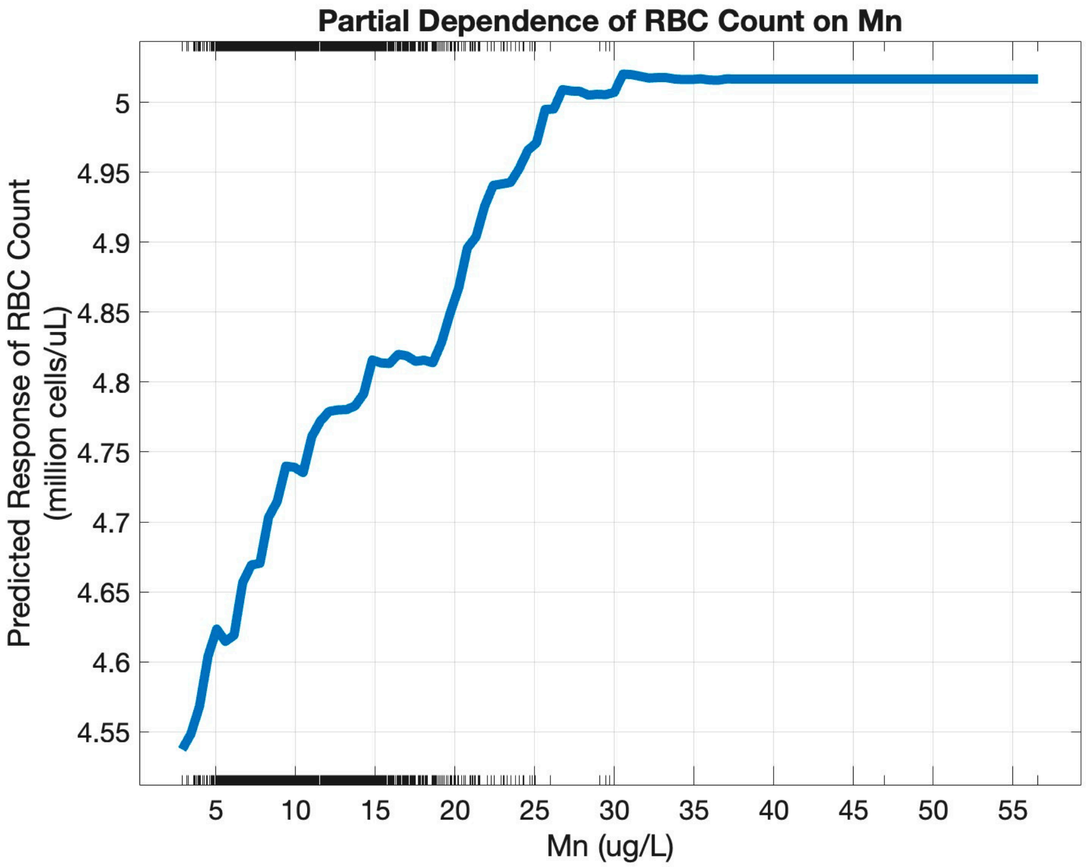


| Optimizable ensemble model |
|---|
| Trained with Cr, Mn, Se, age, sex, ethnicity, income, education, and smoking data |

**Figure S3.** Partial Dependence of RBC count on selenium

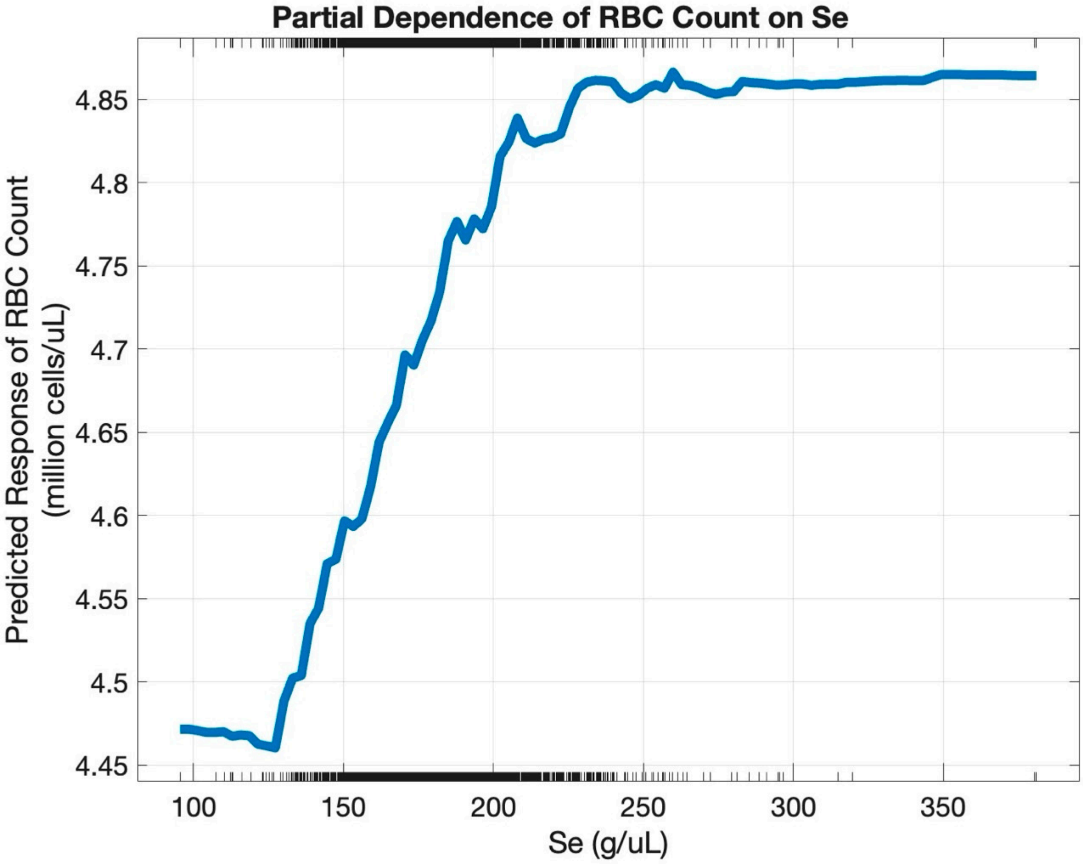


| Optimizable ensemble model |
| --- |
| Trained with Cr, Mn, Se, age, sex, ethnicity, income, education, and smoking data |

**Figure S4.** Partial Dependence of hemoglobin on chromium

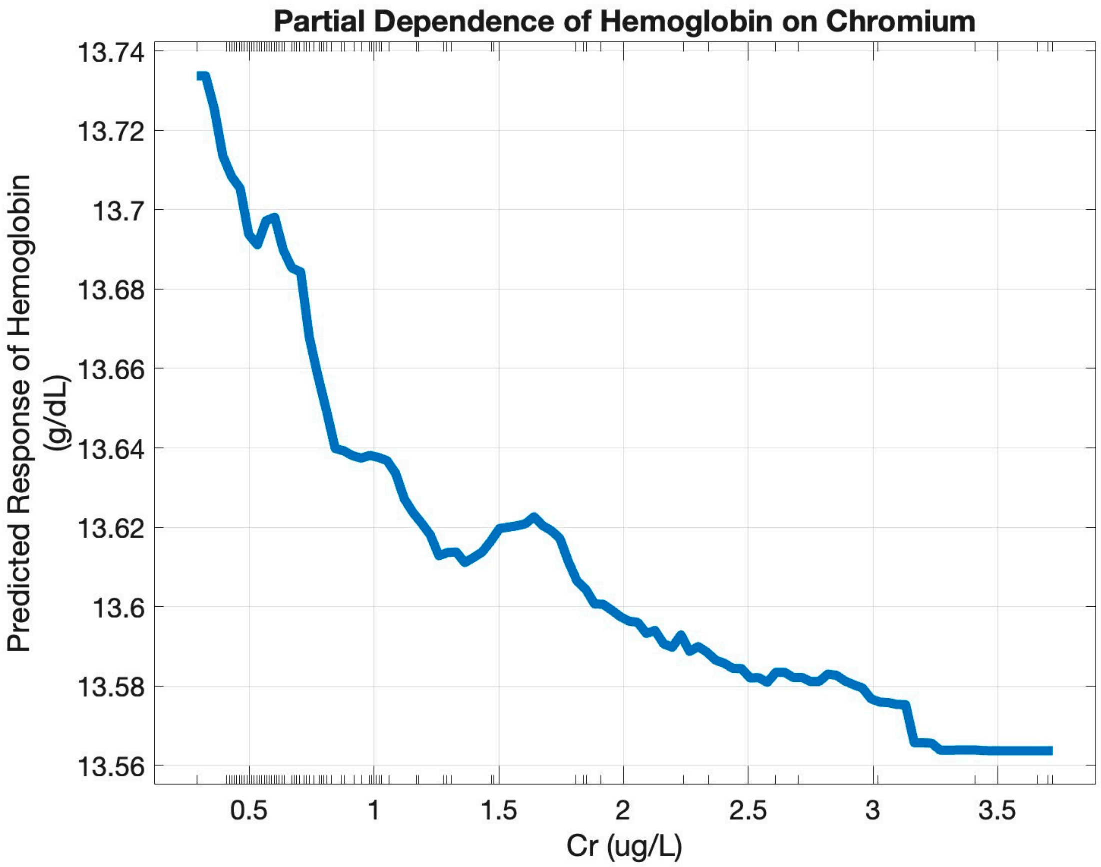


| Optimizable ensemble model |
| --- |
| Trained with Cr, Mn, Se, age, sex, ethnicity, income, education, and smoking data |

**Figure S5.** Partial Dependence of hemoglobin on manganese

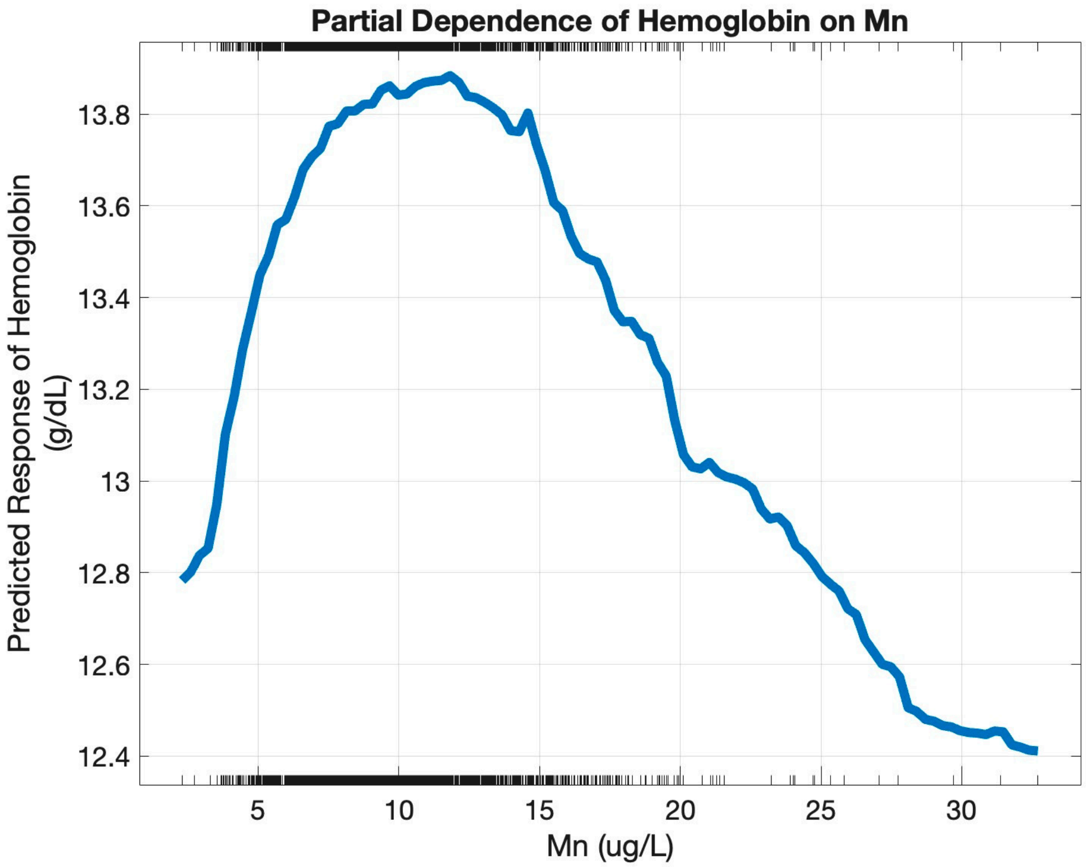


| Optimizable ensemble model |
| --- |
| Trained with Cr, Mn, Se, age, sex, ethnicity, income, education, and smoking data |

**Figure S6.** Partial Dependence of hemoglobin on selenium

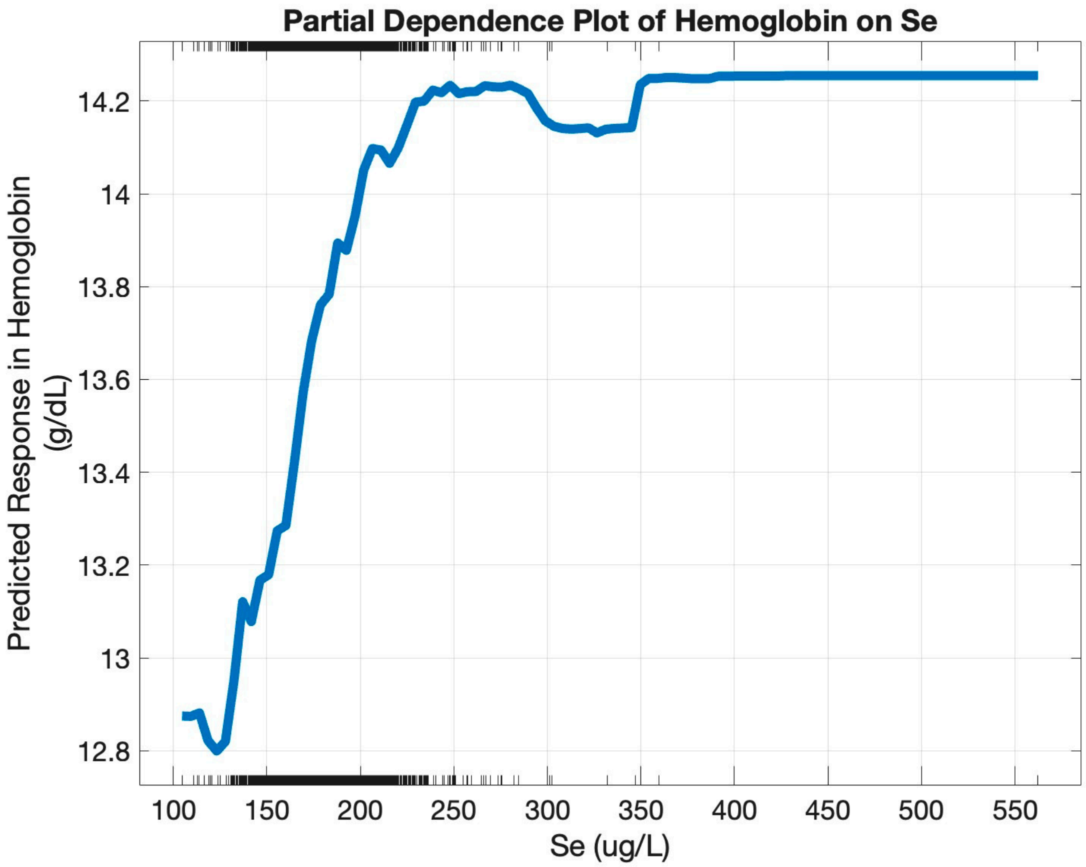


| Optimizable ensemble model |
|---|
| Trained with Cr, Mn, Se, age, sex, ethnicity, income, education, and smoking data |

**Figure S7.** Partial Dependence of hematocrit on chromium

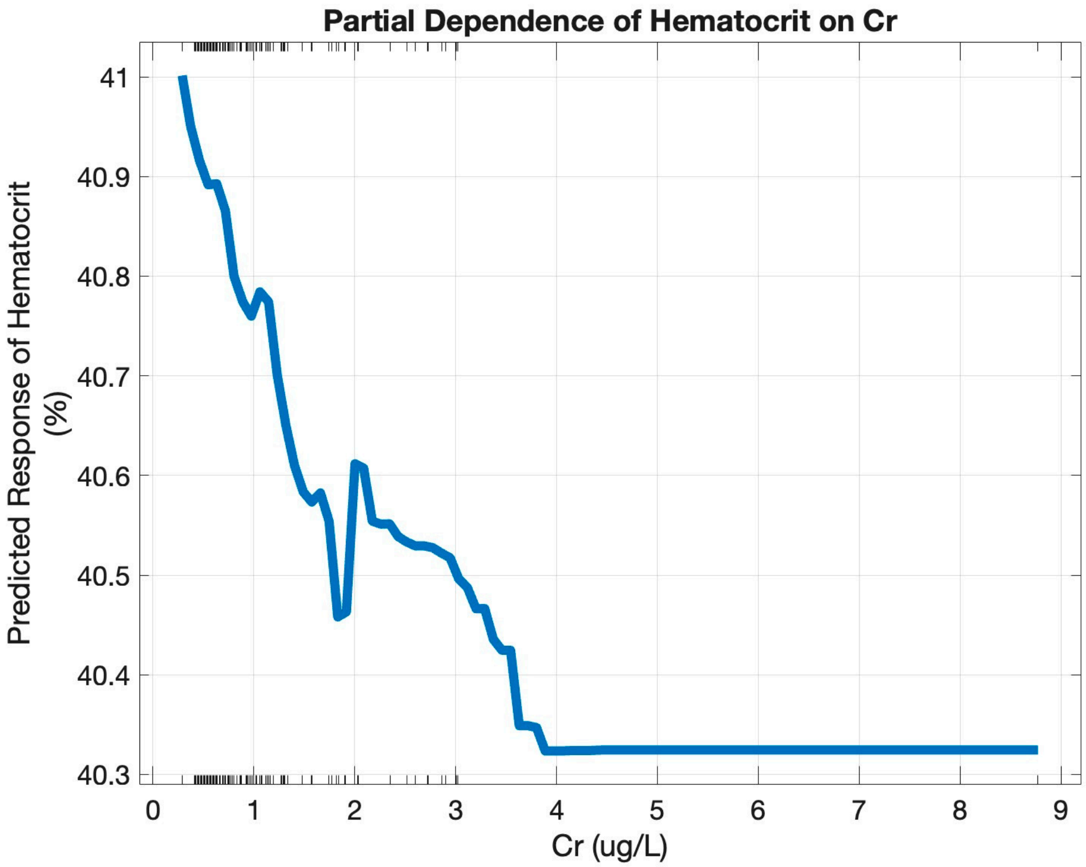


| Optimizable ensemble model |
| --- |
| Trained with Cr, Mn, Se, age, sex, ethnicity, income, education, and smoking data |

**Figure S8.** Partial Dependence of hematocrit on manganese

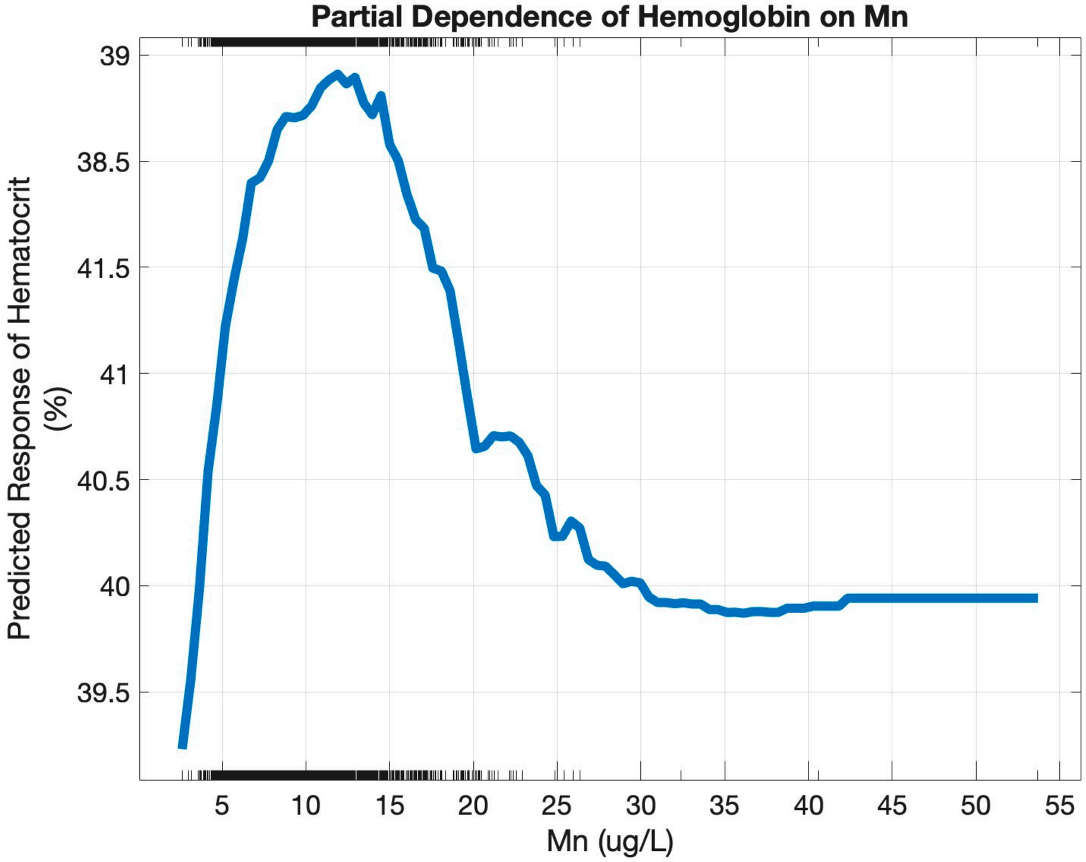


Optimizable ensemble model

Trained with Cr, Mn, Se, age, sex, ethnicity, income, education, and smoking data

**Figure S9.** Partial Dependence of hematocrit on selenium

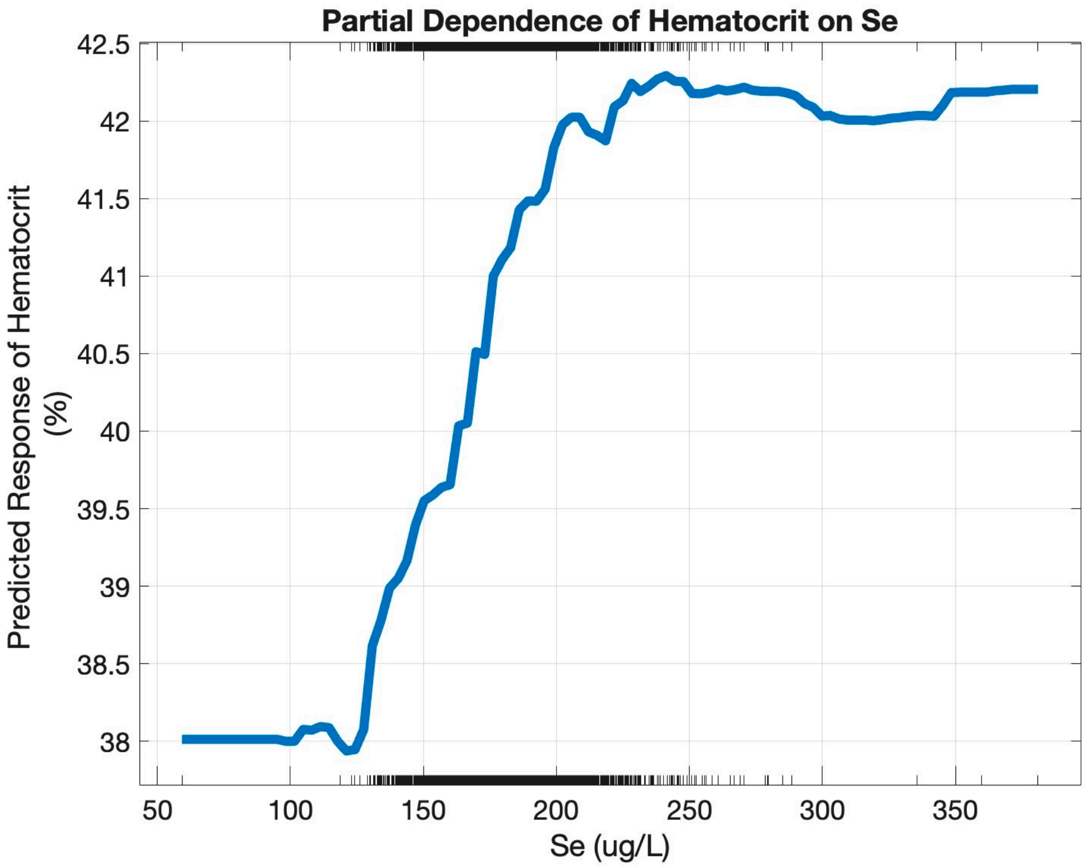


Optimizable ensemble model
Trained with Cr, Mn, Se, age, sex, ethnicity, income, education, and smoking data
